# Hospital and Institutional News

**Published:** 1917-12-08

**Authors:** 


### HOSPITAL AND INSTITUTIONAL NEWS.

### THE MINISTRY OF HEALTH.

A STATEMENT has appeared in some newspapers that a Bill will shortly be introduced to establish a Ministry of Health, with Dr. Addison at its head; it is further stated that it is intended to bring the whole or almost the whole of the medical profession under the new department, and the medical profession will be "nationalised"; so that free medical attendance will be open to all. Such is the very crude scheme which has been described, but how much truth there is in it it is hard to say. A more or less official statement has been issued that it is not true that Dr. Addison has accepted the new post, but there has been no direct denial that the Government has some such idea under consideration. The financial aspect of the scheme is by no means the least important, and a very little thought will show that the expense of any such scheme as that suggested would be enormous and that the present moment, when we are finding great difficulty in financing the war, is not the time to undertake new and almost limitless liabilities such as would be incurred should this scheme come to fruition. With the establishment of a Ministry of Health we are fully in accord, but it will need the greatest care to make it a success, and any hurried legislation can only lead to trouble.

### RATIONS FOR CHILDREN.

SIR ARTHUR YAPP has issued a scale of weekly voluntary rations for children. He has divided the children into four classes, according to age; in the first class he has put children under five years of age; in the second class, those between six and eight years of age; in the third class, those from nine to twelve; and in the fourth class, those from thirteen to eighteen years of age. To all the children half a pound of sugar a week is allowed. The amount of bread increase from 3 lb. in the youngest class to 6 lb. in the oldest. The "other cereals" increase from 6 oz. to 10 oz.; the meat, from 1 lb. to 2 lb.; and fats, including butter and its substitutes, from 6 oz. to 10 oz. The only difference between the third and fourth class is that the latter have a pound and a half of bread more than the former. In the first three classes girls are to have the same amount as the boys, but in the fourth class they may receive four-fifths of the amount allowed to boys of the same age. The intention of this scale of rations is no doubt most excellent, but we should hesitate to guarantee that it is entirely satisfactory. Everyone who has had much to do with the feeding of children knows how enormous are the differences between the appetites of children of the same standing, and even of the same family; and not only the appetite may vary greatly, but the need for food varies greatly also. On the whole, the scale is liberal when compared with the amount allowed to adults. For instance, a boy of thirteen is allowed as much meat, fat, and sugar as any man, and a pound and a half more bread than a sedentary man. If, as seems very likely, compulsory rations become necessary, it will be interesting to see what amounts of food are then allotted to children, for it is of the highest importance that growing children should not be stinted.

### THE HOUSING OF THE PEOPLE.

THE military and political aspects of the war are so pressing and so conspicuous that we are apt to lose sight of other consequences. Many of these latter will only reveal themselves when peace returns and we enter upon the attempt to restore the national life to normal channels. A new page will then be turned, and what will be written there will not be determined solely by precedent and tradition. Never again will things be just what they have been in the past, and persons who have difficulty in accommodating themselves to new situations will be likely to receive some abrupt shocks. For a considerable time, at all events, we shall have to submit to a much wider and more penetrating Government control than was our lot in the past, for this huge convulsion of the war has shaken social life to its foundations. A conspicuous instance of this position is seen in the housing question. Building during the last three years has been almost at a standstill. Yet houses, like other mundane developments, wear out, and the wants of the new generation are ever expanding. When peace returns, capital and labour and materials will all be in demand. Of labour there may be enough and to spare, but of capital and materials who can speak with confidence? A Committee of the Local Government Board which has been appointed to deal with the situation has an enormous task in front of it. Roughly, it is estimated that already the supply of workmen's houses in urban and rural areas is some 200,000 below the requirements of the situation, and the defect will be a progressive one. It is impossible to believe that such a position can be redeemed by private enterprise. With Government or municipal subsidies there must needs be associated some measure of public control, and the building trade will thus necessarily find itself in contact with official regulations. Here is one of the problems which the Ministry of Reconstruction has been constituted to study, and evidently it is of the highest importance that preparations to deal with it are made in advance. For the coming of peace we all ardently pray, but do not let us imagine that it will bring smooth times in its train.

### A MEDICAL OFFICER'S ACTION FOR LIBEL.

A REMARKABLE case involving an assistant medical officer, a matron, and the chairman of the Stockport Board of Guardians has resulted in a verdict for the latter, against whom the medical officer had brought an action for libel. Since the case deserves fuller notice than we had space for last week, we summarise it in the present issue. Dr. Collingwood Fenwick, the plaintiff, after service in France, became resident assistant medical officer at Stepping Hill Hospital, under the Stockport Board of Guardians, in March 1916.

Differences quickly arose between him and the matron, a relative of the defendant, Dr. James Worthington. The plaintiff objected to the matron having the key of the poison cupboard, and to her reviewing the sick orders. He also objected to her being informed of any change in the condition of his patients. His letters to the Board of Guardians enforcing these contentions were at length supported by a series of resolutions in accord with them. Friction, however, did not cease, and the occasion of the alleged libel arose out of the chairman's objection to the attendance of the nurses at the plaintiff's *post-mortem* demonstrations, when, in the presence of the ward, the chairman declared that this proceeding must stop, and that the plaintiff was "non compos mentis" to have permitted it. In the event the occasion was held to be privileged, and the plaintiff's case was dismissed.

### THE MORAL OF THE STEPPING HILL CASE.

THE point of interest however, is not the personal one. It concerns the principles of administration under which Poor-Law medical wards are conducted. We understand that prior to Dr. Fenwick's appointment the Stepping Hill institution had not possessed a resident medical officer, and that the new post was created in part at least because provision was being made for the wounded. Dr. Fenwick in the course of his evidence declared, apparently without contradiction, that the regulations laid down that "lectures should be given to nurses and probationers, and that these included lectures on anatomy and physiology for first-year probationers, and two *post-mortem* demonstrations per session, including dissected parts." On the other hand, in the course of cross-examination Dr. Fenwick agreed that attendance at *post-mortem* examinations was not a necessary part of nurses' training. The matter was the subject of another resolution which decided that "no more *post-mortems* should be held at this military hospital." We suggest that the whole case (which might never have reached publicity if the alleged libel had not been quite unnecessarily repeated) is a criticism on the present haphazard condition of unseparated infirmaries. The regulations and their interpretation are loosely drawn and were inadvisedly administered, and this state of affairs is always liable to arise so long as the sick wards of Poor-Law institutions are not separated from the workhouse, and not placed wholly under medical control.

### THE FETISH OF CENTRALISATION.

IN these days of bureaucratic centralisation the proceedings of the Liverpool Council of Voluntary Aid invite attention, and the friendly criticism of those who know from experience how prone the fetish of charity organisation is to run to seed in that kind of regimentation which is supposed to be peculiar to Prussia. A sign of the times is the hope of the Charity Commissioners that the provisions of the War Charities Act may be extended to all charities. The voluntary hospitals will probably agree that this would eliminate certain undesirable competitors, but they also know that the registration of hospitals would add to the forms which they have to furnish, and they have occasionally been heard to grumble that the time occupied in filling up the returns for "Burdett's Hospitals and Charities" is more than they like to spare! King George's Fund for Sailors offers another instance of the difficulties inherent in centralisation. (We emphasise them because the current cant is that the difficulties are all the other way.) the Fund has been welcomed in Liverpool, but it is pointed out that the local charities in seaport towns may be injured if local annual subscribers are enlisted on behalf of the new central Fund. It is true that the King's Fund met with a similar criticism and survived it, for the feared result did not arise. But the King's Fund was a London Fund. The new Fund must not forget the local problems of the provinces.

### A WORD OF CAUTION ON CORNWALL'S SANATORIUM.

THOSE who understand the gravity of the tuberculosis problem, and who appreciate the real place of the sanatorium in preventive treatment, are suffering from the reaction produced by the anti-phthisis crusade. Like all newspaper questions, the crusade was overdone. There was much noise, some expenditure, and little thought. In consequence the sanatorium degenerated in popular esteem from a fashion to "a fraud," and the problem did not benefit by this want of consideration. In Cornwall, however, the sanatorium is still a cherished scheme, and, in typical English fashion, private and public authorities are combining to provide a county institution. If the purchase money can be raised by voluntary contributions the County Council has agreed to equip and maintain the sanatorium. At a recent meeting at Truro the advantages of adapting Tehidy Mansion were considered. The purchase price is £10,000. Of this a considerable sum has been subscribed. This new sanatorium is to be Cornwall's War Memorial. It is, we believe, the county's first move in this direction, and, since the site appears to be good and sufficient, we need only repeat the classic caution: that all depends upon the choice of a specially qualified medical superintendent. He must have administrative and special experience. He must have had much clinical experience. He must be well read in the Continental literature of his subject, which far exceeds our own. He must be acquainted personally with the tuberculin, pneumothorax, and other special treatments. He must have studied the psychological factors which made Walther's reputation. We emphasise these qualifications because they have hardly ever been insisted on in England. Hence the partial failure of the sanatorium in England.

### WOMEN AS HOSPITAL SECRETARIES.

THE Royal Albert Hospital, Devonport, whose honorary secretary, Captain Dickinson, after nineteen years' service, has been offered a life-governorship of the hospital, is now temporarily employing a woman as his successor. Miss Bradford, who is undertaking the work, has had some previous experience, and is one of those who went out in an administrative hospital capacity to Serbia, whence she has lately returned. After the war it is possible that there will be a reaction from the employment of women in many capacities where they are, sometimes to their hurt, a necessity at present. Are they likely to become competitors for hospital posts? Will the example set by women's hospitals be followed?

### JUBILEE OF THE NEW HOSPITAL FOR WOMEN.

IN 1867 the New Hospital for Women was founded, and its jubilee is being recognised by an attempt to raise a fund sufficient to endow fifty beds. Were this fund raised, half the number of beds would be endowed, and a capital sum would be secured. The appeal is directed mainly to women in the different branches of industry: college students, Civil servants, actresses, gardeners, munition workers, and domestic servants will, it is hoped, endow a bed apiece to commemorate the pioneer work of Dr. Garrett-Anderson, which, women believe, will finally open the door of all professions to them.

### WORKMEN'S CONTRIBUTIONS AT MERTHYR.

RECENTLY the Board of the Merthyr General Hospital appealed to the workmen of the borough to increase their contributions, but after a long discussion the governors of the hospital, at a special meeting on November 15, rejected the terms upon which the workmen offered to subscribe four shillings per man and two shillings per boy per annum to the institution's funds. It was pointed out that if the governors acquiesced in the conditions sought to be imposed, in effect the workmen would obtain entire and absolute control of the hospital. Mr. F. T. James, the treasurer, remarked that the men had the alternative in their own hands, which was to build and maintain a hospital of their own.

### THINKING IN DECIMAL POINTS OF A PENNY.

THE National Health Insurance Commissioners have consented to a slight increase in the rate of payment for the dispensing of medicines under the Insurance Act. It is a common saying that "Chemists think in halfpence"; this, of course, is a slander, but it is quite correct—at any rate technically correct—to say that the Insurance Commissioners in arranging terms with panel chemists think in terms of decimal points of a penny. A year or so ago the Commissioners increased the dispensing fees by 0.3d. per prescription, and now they propose to add a further 0.45d. per prescription, this higher rate to come into force at the beginning of next year. It is estimated that the total sum represented by this extra 0.45d. amounts to something in the neighbourhood of £50,000, a tolerably respectable total, which when divided up among the thousands of panel chemists will not go far in the direction of meeting the increased working expenses of war-time. In notifying the Pharmaceutical Society that the Treasury has given the Commissioners the authority to make this extra payment, the Commissioners state that the granting of the allowance is conditional upon a satisfactory and adequate pharmaceutical service being secured for the ensuing year. A few of the more adventurous spirits among the dispensing chemists protest that the increase is inadequate, but there is no likelihood of a "strike," since the less adventurous ones are in an overwhelming majority.

### THE NEW MEDICAL BOARDS.

THE regulations affecting the examination and grading of men for National Service have been made public in the House of Commons by Sir Donald Maclean. Elaborate precautions have been taken to remove any legitimate grievances, and if the examinee is not satisfied with his grading he can claim re-examination by a National Service Board. Every man not serving with the Colours, or an absentee, can, if he so desires, obtain a medical examination and grading by one of the National Service Boards on application made to the Local National Service Office, formerly the Recruiting Office. This procedure does not, however, in any way affect the man's statutory right to apply for exemption to a Tribunal on any grounds save those of health; but in the case where an appeal is pending, the County of London Appeal Tribunal proposes to adjourn its consideration till the result of the applicant's re-examination is known. It is obviously important that these opportunities shall not be abused so as to delay proceedings, and measures have been taken to ensure that the decisions of the medical assessors shall be promulgated as speedily as possible.

### MATCHES AS A SOURCE OF INCOME.

To sell a cheap article at a high price, in return for supplying a fashionably dressed lady as a saleswoman, is an old device for raising the wind; but, save for strong evidence of its potency, we should not have thought that it would be successful in the case of articles over the commercial price of which everyone is grumbling. Nevertheless, a bevy of women, we understand, lately enlivened the streets of Camberwell selling matches and packets of pins for the benefit of King's College Hospital. Where did the matches come from? And where the pins? Match and Pin Day was devised to help to raise the additional £15,000 a year which has been asked for. We hope that it was successful.

### CHEAP MILK FOR BABIES.

THE increase in the price of milk cannot have other than a harmful effect on the health of infants, for whom milk is a necessity, and local bodies have for some time been endeavouring to find some means of supplying milk at a reasonable price to those who cannot at present obtain it. The Public Health Committee of the Wandsworth Borough Council, acting in conjunction with the local Food Control Committee, proposes to enter into arrangements with the retail dealers to supply milk at the price of 5d. a quart for infants under the age of two years whose parents are unable to pay the present price. The difference between the prices is to be defrayed by the public bodies concerned. It has been estimated that the total cost of carrying out this proposal will be about £7,500 a year, and that the number of children supplied will be about 1,800.

### FORM W. 3635 AND V.A.D. MEALS.

The monthly gazette of the 2nd General Hospital, Manchester, seems to be unique in possessing a page of editorial notes devoted to the critical consideration of hospital and military topics. To go no further back than the October number of the *Searchlight*, as this gazette is aptly called, we find paragraphs recording various changes, with critical comments. For instance, the recent issue of Army Form W. 3635 to V.A.D.s is noted by the typically military form in which the questions are put, whence the inference is drawn that there is some basis for the current rumour that before long we shall see the compulsory enlistment of women. The *Searchlight* argues that equal privileges entail equal responsibilities, and we may add that the latter are not adequately represented by a punctual attendance at the polling booths of Mr. Tweedledum or Mr. Tweedledee. Other notes deal with even more burning matters. They note that " the cutting down of the meat ration from one pound to three-quarters of a pound may have had some justification, even though the meat supplied to detachments is vastly inferior to that provided for patients in hospitals or supplied through shops to the public. . . . If universal rationing will do away with the ' extras ' allowance and place upon the authorities the full responsibility of keeping the troops supplied with the food necessary to enable them to perform their duties efficiently, then its advent will bring general satisfaction." It is a huge relief to find a war hospital gazette taking its readers' interests seriously, and when we do find it at this useful task we have not space to refer to mere jokes and queer drawings.

### THE CRAIGLEITH CHRISTMAS NUMBER.

The Christmas number of the *Craigleith Hospital Chronicle* maintains its custom of containing among contributions from the staff special articles by distinguished outsiders. For instance, in this case, Sir Ernest Shackleton writes an account of an expedition "Across the Antarctic in an Open Boat," which, amid incredible cold and discomfort, led to the crossing of the island of South Georgia. The appeal of the Crusades seems incredible to some people; but it is easier to understand why men made a pilgrimage to a spot hallowed by tradition in an enviable climate than why they go through ice and snow in search of a spot which does not exist. A padre writes on "Humour," and some readable "Nursery Rhymes," which have already been reprinted, help to vary the contents, which include learned articles on the history of the Scottish regiments and a further instalment of historical notes on the Scottish Navy. It will be noticed that the clergy are regular contributors, and this is as it should be, since men of their education should certainly contribute largely to the ephemeral literature of the war.

### NO MORE CASTOR OIL?

Those hospitals who do not hold a large stock of castor oil are likely to be entirely without this valuable medicine very soon. Inquiries made of several leading drug houses have elicited the information that all stocks are held at the disposal of H.M. Government for a special use for which castor oil is particularly suitable. Following on the lack of any glycerine, for which many "substitutes" of doubtful value from the medical standpoint are in vogue, the dearth of castor oil will be severely felt as there does not appear to be at present any efficient substitute to take its place. Castor oil is regarded by many eminent medical men as the most valuable laxative of the Pharmacopœia, and its uses and action for maternity cases and children's ailments are well known. Probably recourse will be had to liquid paraffin, which is now in better supply, and to olive and other vegetable oils, though these are scarce and dear. Our advice to those responsible would be to conserve for the present the stocks they hold, and to use castor oil only in those cases where no other laxative is suitable. It may be that later on a supply will be available, but our experience of the past three years should warn us against counting on this.

### THIS WEEK'S DRUG MARKET.

The upward movement of prices is still noticeable, although the rate of the advance is slower One of the products which is not moving upwards in price is saccharin; supplies are more plentiful, and still larger quantities are expected to arrive from the United States in due course. The demand for saccharin is very large, but the considerable supplies that are expected will probably have the effect of keeping the price down. Honey still tends towards higher prices. Benzoic acid and sodium benzoate are again dearer. The small amount of cocaine available has resulted in still higher prices being asked. Glucose is scarce, and the value has an upward tendency. A fair business is reported in quinine, and stocks are becoming further reduced. Hexamine is dearer, as also is antifebrin. There is a considerable demand for glycerophosphates, and makers are dealing with orders in rotation. Makers of hypophosphites are fairly busy. Paraldehyde is firmer.

### TO OUR READERS.

Contributions are specially invited from any of the readers of The Hospital. They should deal with topical subjects and news or incidents illustrating any department of hospital work, its progress or difficulties. They must be authenticated for the information of the Editor only. The minimum payment if published is 5s. There is no hard-and-fast rule as to space, but paragraphs of about twenty lines in length are preferred.

